# Efficacy and safety of lacosamide in patients with trigeminal neuralgia: an 8-week pilot dose-escalation study

**DOI:** 10.22514/jofph.2025.011

**Published:** 2025-03-12

**Authors:** Pramot Lappichetpaiboon, Somsak Tiamkao, Supanigar Ruangsri, Jarin Paphangkorakit, Waranuch Pitiphat, Teekayu P. Jorns

**Affiliations:** ^1^Department of Oral Biomedical Science, Faculty of Dentistry, Khon Kaen University, 40002 Khon Kaen, Thailand; ^2^Department of Medicine, Faculty of Medicine, Khon Kaen University, 40002 Khon Kaen, Thailand; ^3^Department of Preventive Dentistry, Faculty of Dentistry, Khon Kaen University, 40002 Khon Kaen, Thailand

**Keywords:** Trigeminal neuralgia, Lacosamide, Carbamazepine, Brief pain inventory-facial

## Abstract

**Background**: Trigeminal neuralgia (TN) is a severe neuropathic pain condition in the orofacial region, with carbamazepine recommended as the first-line treatment. Nonetheless, its application is constrained by unfavorable drug responses and side effects. The objective of this research was to assess the effectiveness and safety of lacosamide, a third-generation anticonvulsant, in individuals with TN, and to juxtapose the findings with observational records from recently diagnosed TN patients who underwent carbamazepine monotherapy within the corresponding timeframe. **Methods**: An 8-week flexible dose titration of lacosamide was performed on newly diagnosed 12 TN patients who were divided into two groups: 200 mg/day (n = 5), and 400 mg/day (n = 7). Outcome measures included average pain score, Brief Pain Inventory-facial scores, and side effects. Patients were followed-up at 2, 4 and 8 weeks after baseline. **Results**: The percentage change of pain score at 4-week visit was compared between both lacosamide groups and patients receiving carbamazepine (n = 6) for four weeks during concurrent period. Both lacosamide groups experienced a decrease in pain score at 2-week follow-up, and differences in average pain score reduction were not observed between the two groups across all visits (*p* > 0.05). The mean Brief Pain Inventory-facial score in the lacosamide 200 mg/day group was higher than that in the 400 mg/day group at the 2-week follow-up (*p* = 0.03). Interestingly, the 4-week follow-up revealed that there were no significant variances in pain intensity between the lacosamide and the contemporaneous carbamazepine cohorts (*p* > 0.05). Frequently noted adverse events were mild somnolence (n = 9), slight vertigo (n = 5), and emotional lability (n = 2) without instances of severe adverse drug responses. **Conclusions**: Lacosamide demonstrates potential as a therapeutic option for patients suffering from trigeminal neuralgia. **Clinical Trial Registration**: TCTR20210811002.

## 1. Introduction

Trigeminal neuralgia (TN) is a neuropathic pain disorder characterized by severe, unilateral and episodic pain in the orofacial region supplied by the trigeminal nerve [1]. The high intensity pain in TN attacks could severely interrupt daily activities and substantially reduce oral health-related quality of life. While there have been advancements in the development of newer medications for treating neuropathic pain, carbamazepine (CBZ) and its derivative, oxcarbazepine (OXC), remain the primary recommended first-line medications for TN [2, 3]. However, the use of CBZ and OXC is limited due to the potential for severe cutaneous drug reactions, which have been reported to be more common in East and Southeast Asian populations due to higher frequency of HLA-B*1502 allele [4, 5]. Additionally, research suggests that at least one adverse drug reaction (ADR) occurs in 70% of patients receiving CBZ therapy [6]. Therefore, non-medication alternatives for managing TN has been found to be beneficial in improving the quality of life through treatments such as acupuncture, laser therapy and neurosurgical procedures [7].

Lacosamide (LCM), a third-generation antiepileptic drug, inhibits voltage-gated sodium ion channels (NaV 1.3, NaV 1.7 and NaV 1.8) during the slow inactivation phase, leading to a prolonged depolarization period compared to CBZ [8]. In case studies and controlled trials, the medication has been employed as a stand-alone treatment for a range of chronic pain disorders, including but not limited to painful diabetic neuropathy [9, 10], small fiber neuropathy [11] and short-lasting unilateral neuralgiform headache attacks with conjunctival injection and tearing (SUNCT) [12]. There is no universally agreed-upon dosage of LCM for managing TN. However, case reports have shown that dosages in the range of 50–600 mg/day have been effective in treating refractory TN, TN caused by intracranial tumors, and persistent idiopathic facial pain [13, 14]. Furthermore, studies have shown that administering 150 to 200 mg of LCM through intravenous infusions lasting 30 to 40 minutes can effectively alleviate acute pain episodes and is better accepted by patients with trigeminal neuralgia when compared to phenytoin [15]. However, the antineuralgic effect of LCM as a monotherapy, or in comparison with CBZ, has yet to be evaluated in a prospective controlled trial involving TN patients.

Our study aimed to assess the effectiveness and safety of LCM at 200 and 400 mg/day in TN patients in a prospective manner. Furthermore, we sought to analyze these findings in contrast to observational data collected from newly diagnosed TN patients who received CBZ monotherapy during the corresponding timeframe.

## 2. Materials and methods

### 2.1 Study design and setting

This study was approved by the Center for Ethics in Human Research, Khon Kaen University, Thailand in accordance with the Declaration of Helsinki and the International Conference on Harmonization Good Clinical Practice (ICH GCP) Guideline. An 8-week, non-randomized, prospective, controlled pilot clinical trial was conducted in all consecutive TN patients seeking examination and treatment at the Orofacial Pain and Oral Medicine Clinic, Faculty of Dentistry, Khon Kaen University, Thailand, between April and December 2021. Inclusion criteria consisted of newly diagnosed TN patients identified by an orofacial pain specialist (TPJ), all of whom met the diagnostic criteria for classical trigeminal neuralgia according to The International Classification of Headache Disorders 3rd edition (ICHD-3) with a pain score of 4 or higher on the numeric rating scale, and who had not previously received any treatments for TN or experienced pain after the natural remission period. Patients with complex physical or psychological conditions, additional neuropathic pain, significant liver or kidney dysfunction, inability to attend follow-up appointments, or a lack of willingness to take part were considered as exclusion criteria. Additionally, the study also included new TN patients meeting the same criteria who were receiving CBZ treatment at the same time.

### 2.2 Intervention

After providing a comprehensive explanation of the study and obtaining informed consent, patients underwent pretreatment evaluations, which included a complete blood count, liver enzyme tests (alanine transaminase and aspartate transaminase), creatinine assessment and human leukocyte antigen B gene (HLA-B*1502 allele) testing. Subsequently, patients were administered LCM (7535603, Vimpat® 100 mg/tablet, Aesica Pharmaceuticals GmbH, Zwickau, Germany) with a fixed-dose titration up to 200 mg/day during the initial 2-week phase. Follow-up visits were scheduled at 2, 4 and 8 weeks after the baseline assessments. To address breakthrough exacerbations of pain during the study period (as assessed by the patients, indicating exacerbation that did not respond to LCM treatment), patients were informed about the option of rescue medication, specifically gabapentin, prescribed at dosages ranging from 300 to 1200 mg per day. None of the participants in our study needed to use gabapentin as a supplementary treatment for worsening pain during the entire research period. At the initial 2-week follow-up appointment, the patients were divided into two groups according to their symptom improvement and personal preference: those who continued with a daily dose of 200 mg and those who opted to increase it to 400 mg. The selected LCM dosage was then consistently maintained throughout the remaining study period, as illustrated in Fig. 1.

**Fig. 1.** Study flow diagram. CBZ: Carbamazepine; NRS: Numeric Rating Scale; BPI-facial: Brief Pain Inventory-facial; CBC: Complete Blood Count; LFT: Liver Function Tests; LCM: Lacosamide.

To assess the pain control efficacy of LCM in comparison to standard medication CBZ, observational pain scores of new TN patients concurrently undergoing CBZ treatment, with a gradual titration to 200–400 mg/day over a four-week duration were documented. Pain scores were measured both prior to the initiation of treatment and after four weeks of CBZ administration.

### 2.3 Outcome measurements

Study outcomes were measured at baseline and during each follow-up visit and collected by the dental assistants. The primary outcome measure was the change in average pain intensity, assessed using a numeric rating pain scale ranging from 0 (pain-free) to 10 (worst imaginable pain). Patients assessed their pain levels at the pretreatment visit, as well as during the 2-week, 4-week and 8-week follow-up visits. The reduction of average pain score more than 3 was considered as a clinically meaningful change.

The secondary outcome included the Brief Pain Inventory-facial (BPI-facial) score and the assessment of adverse effects. The BPI-facial survey was comprised of two sections: one focusing on general activity disruptions and the other on activity disruptions specific to facial expressions [16]. Each component comprised questions about various activities affected by facial pain, rated on a numerical rating scale from 0 (no interference) to 10 (complete interference).

To monitor adverse effects, blood samples were collected for the analysis of complete blood count, electrolytes (calcium, sodium, potassium and phosphate), liver function (alanine transaminase and aspartate transaminase) and kidney function (creatinine). Patients were also directed to keep daily pain diary booklets provided at each visit. These booklets were collected at the subsequent appointment to document their medication adherence, side effects and any adverse reactions experienced.

Following the completion of the study, patients may explore the option of either continuing LCM as a treatment or transitioning to CBZ, the established standard medication, in the absence of the HLA-B*1502 allele. Additionally, the possibility of undergoing microvascular decompression surgery to potentially cure the condition was recommended.

### 2.4 Statistical analyses

The analysis was conducted using SPSS Statistics version 22 (IBM Corp., Armonk, NY, USA). The characteristics of the two LCM groups and the CBZ group were compared using Fisher’s Exact test for categorical variables and the Kruskal-Wallis test for continuous variables. Pain scores and BPI-facial scores of the two LCM groups were compared at each visit using the Mann-Whitney U test with Bonferroni adjustment. The percentage change in pain score during the fourth week of the study for the two LCM groups and the CBZ group was compared using the Kruskal-Wallis test.

## 3. Results

Twenty-five new TN patients were initially screened, and 13 patients were eligible to receive LCM. However, one patient dropped out of the study due to unsatisfactory pain control before the 2-week follow-up. The study flow diagram is presented in Fig. 1. The demographic and clinical data at baseline of the patients who received 200 mg/day and 400 mg/day of LCM, as well as the 6 patients who received CBZ, are shown in Table 1. Although there was a slightly lower average age in the LCM 200 mg/day group compared to the 400 mg/day and CBZ groups, there were no statistically significant differences among the three comparison groups (*p *> 0.05).

**Table 1.** Demographic and clinical data of the patients at baseline.

		Lacosamide 200 mg/d (n = 5)	Lacosamide 400 mg/d (n = 7)	Carbamazepine 200–400 mg/d (n = 6)	*p*-value
Gender (%)					
	Male	1 (20.0%)	3 (42.9%)	2 (33.3%)	0.83^a^
	Female	4 (80.0%)	4 (57.1%)	4 (66.7%)	
HLA-B*1502 Pharmacogenetics (%)					
	Positive	2 (40.0%)	1 (14.3%)	0 (0.0%)	0.20^a^
	Negative	3 (60.0%)	6 (85.7%)	6 (100.0%)	
Age in years					
	Mean (SD)	55.6 (4.1)	63.4 (2.2)	62.3 (7.0)	0.37^b^
	Range	49–65	53–67	38–74	
Mean pain score (SD)		7.0 (1.1)	7.9 (0.9)	7.2 (1.6)	0.74^b^
Affected CN V branches (%)					
	V2	3 (60.0%)	3 (42.9%)	3 (50.0%)	0.36^a^
	V3	0 (0.0%)	2 (28.6%)	3 (50.0%)	
	V2, V3	2 (40.0%)	2 (28.6%)	0 (0.0%)	
Affected side of face (Frequency, %)					
	Left	2 (40.0%)	2 (28.6%)	3 (50.0%)	0.83^a^
	Right	3 (60.0%)	5 (71.4%)	3 (50.0%)	

*^a^Fisher’s Exact test; ^b^Kruskal-Wallis test. SD: Standard Deviation; CN: Cranial Nerve.*

### 3.1 Pain reduction

Fig. 2 presents a comparison of average pain scores between 200 mg/day and 400 mg/day groups at various visits. During the pretreatment visit, the mean pain score of the 400 mg/day group was slightly higher than that of the 200 mg/day group. Notably, patients in the 200 mg/day group experienced a marked decrease in pain score at the 2-week follow-up visit, after which their score leveled off at the 4-week visit (2.6 and 2.0, respectively). On the contrary, participants in the 400 mg/day category exhibited a more gradual reduction in pain rating during the assessment at 2 weeks, before eventually aligning with those in the 200 mg/day group by the evaluation at 4 weeks (registered at 6.1 and 3.3 correspondingly). The average pain score slightly rose for both groups come the 8-week mark (measuring at 3.2 for the 200 mg/day group and 4.1 for the 400 mg/day group). Noteworthy is the absence of any notable contrast in pain scores between the two groups across the various assessment periods (*p *> 0.05). The mean and median average pain scores of all three groups at each follow-up period are shown in Table 2.

**Fig. 2.** Comparison of mean pain score between lacosamide 200 mg/day and 400 mg/day groups.

**Table 2.** Comparison of pain scores among the lacosamide and carbamazepine groups at each visit.

Pain score		Lacosamide 200 mg/d (n = 5)	Lacosamide 400 mg/d (n = 7)	Carbamazepine 200–400 mg/d (n = 6)	*p-*value
Week 0					
	Mean (SD)	7.0 (2.5)	7.9 (2.4)	7.2 (1.6)	0.74^a^
	Median (IQR)	8.0 (4.5–9.0)	9.0 (5.0–10.0)	9.0 (3.8–10.0)	
Week 2					
	Mean (SD)	2.6 (2.1)	6.1 (2.7)	Data not available	0.14^b^
	Median (IQR)	2.0 (1.0–4.5)	5.0 (5.0–9.0)		
Week 4					
	Mean (SD)	2.0 (1.9)	3.3 (1.80)	1.2 (1.3)	0.13^a^
	Median (IQR)	3.0 (0.0–3.50)	3.0 (2.0–4.0)	1.0 (0.0–2.25)	
Week 8					
	Mean (SD)	3.2 (3.8)	4.1 (1.5)	Data not available	0.43^b^
	Median (IQR)	2.0 (0.0–7.0)	4.0 (3.0–6.0)		

*^a^Kruskal-Wallis test; ^b^Mann-Whitney U test with Bonferroni adjustment. SD: Standard Deviation; IQR: Interquartile Range.*

### 3.2 Physical function scores

The average BPI-facial questionnaire scores for both general and specific physical functions are presented in Table 3. The general physical function disturbance scores showed a decrease in both groups throughout the study period. Although there were statistically significant differences between the two LCM groups in both general and specific physical function scores at the 2-week follow-up visit (*p* = 0.03), the differences at the 4-week and 8-week follow-up visits were not statistically significant (*p *> 0.05). Fig. 3 illustrates the overall and individual physical function impairment scores of both groups throughout the research period.

**Table 3.** Comparison of BPI-facial scores between the LCM groups at each visit.

BPI-facial		Lacosamide 200 mg/d (n = 5)	Lacosamide 400 mg/d (n = 7)	*p*-value^a^
General physical function				
Week 0				
	Mean (SD)	3.3 (1.3)	4.7 (2.0)	0.14
	Median (IQR)	3.4 (2.1–4.4)	5.0 (2.9–5.3)	
Week 2				
	Mean (SD)	0.9 (1.4)	3.4 (1.8)	0.03
	Median (IQR)	0.3 (0.2–1.9)	3.1 (1.6–4.3)	
Week 4				
	Mean (SD)	0.6 (1.4)	2.1 (1.2)	0.73
	Median (IQR)	0.0 (0.0–1.6)	2.1 (1.0–2.9)	
Week 8				
	Mean (SD)	0.9 (1.8)	2.0 (1.9)	0.73
	Median (IQR)	0.1 (0.0–2.2)	1.3 (0.6–3.3)	
Specific physical function				
Week 0				
	Mean (SD)	5.0 (3.4)	7.0 (2.0)	0.34
	Median (IQR)	5.1 (1.8–8.0)	7.0 (4.9–8.6)	
Week 2				
	Mean (SD)	2.4 (2.6)	6.2 (2.4)	0.03
	Median (IQR)	0.9 (0.8–4.8)	5.6 (4.3–9.1)	
Week 4				
	Mean (SD)	1.3 (0.9)	2.5 (1.5)	0.1
	Median (IQR)	1.1 (0.4–2.1)	3.0 (0.7–3.4)	
Week 8				
	Mean (SD)	1.6 (1.7)	3.3 (2.3)	0.2
	Median (IQR)	1.1 (0.6–2.9)	2.4 (1.7–4.6)	

*^a^Mann-Whitney U test with Bonferroni adjustment. SD: Standard Deviation; IQR: Interquartile Range.*

**Fig. 3.** Comparison of mean physical function scores: (A) general physical function disturbance score and (B) specific physical function disturbance score from the Brief Pain Inventory-facial questionnaire.

### 3.3 Percent of reduction in average pain scores

Fig. 4 depicts the percentage change in average pain scores over the 4-week period for the three comparison groups. The percentage of pain reduction was comparable between the two LCM groups (61.25% in the 200 mg/day group and 70% in the 400 mg/day group). The most significant decrease in pain was noted in the 6 patients with trigeminal neuralgia who were administered CBZ (75%), despite the absence of statistically significant variances between the different groups (*p* = 0.65).

**Fig. 4.** Percent reduction in pain scores over a 4-week period in lacosamide 200 mg/day group, 400 mg/day group and carbamazepine group.

### 3.4 Safety

The most observed side effects were mild sleepiness (n = 9), mild dizziness (n = 5) and mood instability (n = 2). No severe adverse drug reactions were reported. There was no statistically significant difference in the occurrence of side effects between the two groups (*p *> 0.05). It is noteworthy that the three patients who tested positive for the HLA-B*1502 allele did not experience any severe cutaneous drug reactions or develop a maculopapular rash.

The results of the blood tests, which included a full blood count, electrolyte levels, and liver and kidney function, were all found to be within the normal range for the majority of patients, with the exception of one individual in the group receiving LCM 200 mg/day. This particular case exhibited elevated levels of alanine transaminase (70 IU/L, normal range 0–25 IU/L) and aspartate transaminase (50 IU/L, normal range 0–32 IU/L) in the 8-week follow-up visit. However, both liver enzyme levels returned to the normal range within 1 month after the patient stopped taking the medication.

Furthermore, there were no changes in the blood test results in the 8 patients who had slightly abnormal complete blood count, liver enzyme levels or serum creatinine from the baseline.

## 4. Discussion

The purpose of this study is to evaluate the antineuralgic effect of LCM in patients with TN. The efficacy and safety of LCM were observed in both the 200 mg/day and 400 mg/day groups throughout the entire study. The pain-relieving properties of LCM demonstrated in this study were comparable to the findings of two prior studies involving individuals suffering from painful diabetic neuropathy and small fiber neuropathy [11, 17]. However, the median pain score of patients in this study slightly increased at the 8th week of the study, which may be related to the natural characteristics of TN, characterized by fluctuations in symptoms. The fluctuations manifest as a cycle of exacerbation followed by natural remission, affecting approximately 53.4% of all TN patients [18].

Although the exact mechanism of action beyond sodium ion channels is unclear, the onset of pain inhibition with LCM may be slower than that of conventional sodium channel blockers due to changes in the permeability of sodium channels. Conversely, CBZ demonstrates biphasic inactivation patterns via ligand gate control during the rapid inactivation phase of sodium channels [8]. After being treated for 12–15 days, more than half of the patients in both groups showed a positive response rate, which is consistent with the findings of Tremont-Lukats *et al*. [19]. Their research indicated that the response rates for patients with neuropathic pain who received CBZ treatment ranged from 70%–89% within 5 to 14 days.

LCM demonstrated a comparable onset of pain inhibition to CBZ, as the percent change in pain score in this study showed no significant difference between the LCM 200 mg/day, 400 mg/day and CBZ 200–400 mg/day groups after 4 weeks of treatment. The comparison of the LCM data from this study with data from patients who received CBZ during the same period may suggest the comparable effects of LCM to standard medication for pain management in TN. Nevertheless, an analysis of past cases involving the administration of oral lacosamide at dosages ranging from 50 mg to 600 mg per day to treat refractory TN saw pain reduction in around two-thirds of the cases after the initial three months of treatment. Notably, there was no substantial escalation in pain relief when lacosamide was used in combination with prior medications like CBZ or OXC [14].

This study aims to evaluate pain according to the Initiative on Methods, Measurement and Pain Assessment in Clinical Trials (IMMPACT) guidelines [20] and to assess the physical functions of patients after receiving treatment. The selection of the BPI-facial measure was based on a systematic review of treatment outcomes in studies on trigeminal neuralgia [21]. Improvement in physical functions was observed in both groups of patients receiving different doses of LCM. Although the BPI-facial score at the 2-week follow-up visits in the LCM 400 mg/day group was significantly higher than in the LCM 200 mg/day group, both general and specific physical function disturbance scores were reduced in both groups at the 4-week visit and the difference was not significant. The group receiving 400 mg/day showed a higher score for physical function disturbance, which could be linked to a reduced response to the medication prompting a dosage increase during the 2-week evaluation. Additionally, there was a slight rise in physical function disturbance scores during the 8-week follow-up, corresponding with an increase in pain ratings.

The adverse effects of LCM were dose-dependent and showed no significant differences when compared to CBZ [14, 22, 23]. There were no differences in the side effects in both patient groups treated with LCM in this study. Common side effects such as sleepiness, dizziness, nausea and loss of appetite were reported. Dividing the medication into two doses per day could help reduce these side effects [24]. While most of the patients did not experience severe side effects or systemic complications after taking LCM, one patient receiving 200 mg/day LCM exhibited elevated liver enzymes after four weeks of maintained dose. After stopping LCM, the levels of liver enzymes in the patient reverted to normal, aligning with a documented instance of liver toxicity in a patient with epilepsy [25]. Adverse effects may be related to LCM metabolism through cytochrome CYP2C19, CYP2C9 and CYP3A4. Some concomitant drugs, such as warfarin, omeprazole or digoxin, may alter the metabolism process, leading to an increase in LCM serum levels. Hence, it is recommended that patients with slightly to moderately damaged liver function should not surpass a daily dosage of 300 mg of LCM [26].

Among the 3 patients who tested positive for the HLA-B*1502 allele, none experienced severe cutaneous drug reactions or maculopapular rashes. Although there have been reports of severe cutaneous adverse effects in patients receiving LCM and concerns about the possibility of cross-allergic reactions, no clear correlation between adverse reactions to LCM and pharmacogenetic factors has been reported. Furthermore, the relationship between HLA-B*1502 and the potential cross-reactivity in cutaneous drug reactions induced by antiepileptic drugs with LCM has not been definitively established [27, 28, 29].

Furthermore, comparing the pain scores to retrospective observational data from TN patients with similar demographic and clinical characteristics who received CBZ treatment for 4 weeks showed that the percent reduction in pain scores from baseline did not significantly differ among the three groups. By comparing the results, the study’s credibility is reinforced, indicating that LCM may be a promising treatment approach for TN.

The strength of this study lies in its prospective controlled open-label clinical trial design, which included newly diagnosed TN patients who tested positive for the HLA-B*1502 allele. The “proof-of-concept” study design during the forced titration period followed an enrich enrollment strategy, aiming to exclude patients from the study based on factors such as dissatisfaction, poor response, or complications to placebo. This method aids in minimizing discrepancies among patients and enables a targeted evaluation of LCM’s effectiveness in all TN patients [30]. In order to accomplish this goal, a flexible approach to dose titration was utilized, wherein adjustments to the dosage were guided by factors such as patient satisfaction, responses to pain management and the presence of any adverse effects, as opposed to adhering to a rigid dose titration schedule. This approach offered the advantage of higher patient compliance and reduced dropout rates.

The limitations of this study were the limited sample size due to the rarity of the condition, the flexible titration protocol in which patients who had poor response to 200 mg/day dose would receive 400 mg/day could lead to confounding by indication and the highest optimum dose range evaluation could not be performed due to the patients’ responses to low dose of LCM. Furthermore, an 8-week duration for the study may not encompass the natural periods of remission of the disease. More comprehensive research involving multiple centers, extended follow-up periods and randomized controlled trials, with comparisons against placebos or other commonly used medications, would yield stronger evidence. Furthermore, assessing the timing of the pain-relief effects of LCM could enhance our insights into its practical value for individuals suffering from trigeminal neuralgia.

## 5. Conclusions

The administration of LCM at dosages of 200 and 400 mg/day exhibited beneficial analgesic outcomes and enhanced physical performance, while causing minimal adverse effects and devoid of any serious drug responses. Consequently, lacosamide exhibits potential as an alternative standalone treatment for individuals facing challenges with conventional medications, especially those intolerant to the side effects of carbamazepine or possessing the HLA-B*1502 allele. Further randomized controlled trials comparing LCM with placebo or other first-line drugs are recommended.
